# On the tunneling time of ultracold atoms through a system of two mazer cavities

**DOI:** 10.1038/s41598-018-19468-x

**Published:** 2018-01-30

**Authors:** Fazal Badshah, Guo-Qin Ge, Muhammad Irfan, Sajid Qamar, Shahid Qamar

**Affiliations:** 1 School of Physics, Huazhong University of Science and Technology, 1037 Luoyu Road, Wuchang, Wuhan 430074 P. R. China; 2 Quantum Optics Lab. Department of Physics, COMSATS Institute of Information Technology, Islamabad, Pakistan; 3 Department of Physics and Applied Mathematics, Pakistan Institute of Engineering and Applied Sciences, Nilore, Islamabad, 45650 Pakistan; 4 Kavli Institute of NanoScience, Delft University of Technology, Lorentzweg1, 2628 CJ Delft, The Netherlands

## Abstract

We study the resonant tunneling of ultraslow atoms through a system of high quality microwave cavities. We find that the phase tunneling time across the two coupled cavities exhibits more frequent resonances as compared to the single cavity interaction. The increased resonances are instrumental in the display of an alternate sub and superclassical character of the tunneling time along the momentum axis with increasing energies of the incident slow atoms. Here, the intercavity separation appears as an additional controlling parameter of the system that provides an efficient control of the superclassical behavior of the phase tunneling time. Further, we find that the phase time characteristics through two cavity system has the combined features of the tunneling through a double barrier and a double well arrangements.

## Introduction

The most basic system in quantum optics is essentially a two-level atom interacting with a single mode quantized field as realized in a high quality micromaser cavity. In an important study related to the ultraslow atomic interactions with a micromaser cavity, Scully *et al*. realized that such atoms exhibit a non-zero reflection probability even in the absence of any photon inside the cavity. This exclusively new phenomenon in which the cavity acts like a potential to the incident slow atoms^1–4^ was named as Mazer. It is a device for the cold atoms where the microwave amplification is achieved via the z-motion-induced emission of radiation. The potentials experienced by the ultracold atoms due to vacuum cavity modes are usually referred as vacuum induced potentials that are drastically different from those caused by the non resonant fields^5^. For the realization of the Mazer action, one has to cool the atoms to a temperature as cool as 100 nano kelvin. Fortunately, the newly developed laser cooling techniques provide a great opportunity to obtain ultracold atoms whose interaction with the quantized electromagnetic field has drawn a significant zeal of attention^6–10^.

In an interesting study, Agarwal and Arun studied the transmission characteristics for a system of two Mazer cavities^11^. They found that the center of mass (c.m.) motion of the interacting cold atoms couple the two initially uncoupled cavities. The vacuum-cavity induced potentials encountered by the slow atoms can be controlled by the cavity dimensions and their mutual separation. A multi-cavity system may be instrumental in the realization of several concepts in the field of semiconducting superlattices^12^ in an entirely different set-up of the atom-cavity interaction. It is important to note that in a system of high quality microwave cavities, the potential experienced by the incident atoms is quite complex as it modifies with the dressed state of the system.

Quantum tunnelling is a well established feature of the probabilistic nature of quantum mechanics. Among the various definitions of tunnelling time^13–15^, phase time is the most extensively studied both in theory and experiments^16–21^. Recently, phase time has been studied for the tunnelling of ultraslow atoms through vacuum cavity modes^22^. In some prior studies, we have shown that the dark states in multi-level atoms, atomic coherence, off-resonant interactions and mode profile of the cavity strongly modifies the tunneling time through a single cavity^23–27^. Earlier, Recami and collaborators analyzed the problem of tunneling time of non relativistic particle through multiple barriers^28–30^. For the non-resonant tunneling they found that the traversal time is independent of the length of the barrier^29^. A comparison of their theoretical results of the phase time with the experimental data obtained for the neutron tunneling through two barriers show a satisfactory matching of the results^30^.

In this paper, we studied phase time for the resonant tunneling of ultraslow atoms traversing a system of two high-Q Mazer cavities. As compared to the earlier study^11^ which is related to the behavior of the probability of transmission of ultracold atoms, here our main focus is to study the dynamics of tunneling time or traversal time of ultracold atoms. It is an important problem due to its close resemblance to a very well studied phenomenon of the tunneling of electrons though multi barrier potentials^31–36^. We considered that atoms in the initial excited state interact with the cavities prepared in their initial vacuum state. Here, we notice that the tunneling time through the cavities exhibits both sub and superclassical traversal behaviors. It is interesting to see that the intercavity separation plays a crucial role in controlling the superclassical nature of tunneling. In case of a multi-cavity system, due to more frequent resonances in the phase time curve one may realize its alternate sub and superclassical character with increasing energies of the incident atoms. It is useful to mention that effects of the increased resonances in our system is somewhat similar to the Tsu *et al*. findings in case of the resonant tunneling of electrons through semiconductor multi-barrier potentials^31^. In a comparison with the tunneling through double barrier and double well potentials, we find that the tunneling time of the ultracold atoms through the double cavity system has the combine features that are shown either by the double barrier or the double well arrangements.

## Model

**Figure 1:**
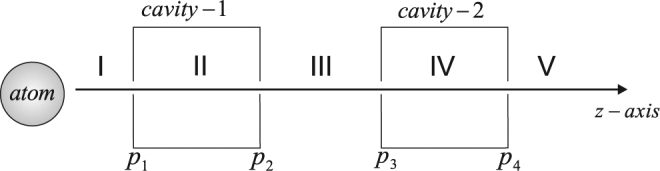
Sketch of our model for the tunneling of ultraslow atoms traversing a multi-cavity system.

We considered ultraslow atoms along *z* axis which interact with a system of two Mazer cavities as sketched in Fig. 1. We take $|e\rangle$ and $|g\rangle$ as the two electronic transition states of the interacting ultraslow atoms. The flux of the incident atoms is so adjusted that no cooperative effects are taking place in the interaction region. Such an interaction may result in the transmission or reflection of the ultracold atoms in either of the two transition levels. Here we take the simple case by considering that the two modes of the cavities are in resonance with the Bohr’s frequency *ω* of the interacting atoms. For the ultraslow atoms, we need to deal with the c.m. motion quantum mechanically. Further, using the dipole and rotating wave approximations the subsequent interaction Hamiltonian take the form^11^

1 $$\begin{array}{rcl}H & = & \hslash \omega |e\rangle \langle e|+\hslash \omega ({a}_{1}^{\dagger }{a}_{1}+{a}_{2}^{\dagger }{a}_{2})+\frac{{p}_{z}^{2}}{2m}+\hslash {\chi }_{1}{u}_{1}(z)\,({\sigma }^{+}{a}_{1}+{\sigma }^{-}{a}_{1}^{\dagger })\\  &  & +\hslash {\chi }_{2}{u}_{2}(z)\,({\sigma }^{+}{a}_{2}+{\sigma }^{-}{a}_{2}^{\dagger }).\end{array}$$

This Hamiltonian contains the term ${p}_{z}^{2}\mathrm{/2}m$, which reveals the effectiveness of the c.m. energy to the atom-field interaction process. We denote mass of the atoms by *m* and destruction, creation operators of cavity 1 (2) by ${a}_{1},{a}_{1}^{\dagger }({a}_{2},{a}_{2}^{\dagger })$. Similarly, the atom-field coupling strengths and mesa mode profiles of cavity 1(2) are given by *χ*_1_(*χ*_2_) and *u*_1_(*u*_2_), respectively. The operators *σ*^−^ and *σ*^+^ represent a sort of transition between the two electronic levels of the interacting ultracold atoms. The mesa mode consideration of the field describes the case where atom-field coupling has a uniform value inside the cavity and it drops to zero elsewhere. Now the collective wave function for our system can be written as

2 $$|{\rm{\Psi }}\rangle ={{\rm{\Phi }}}_{0}|e\mathrm{,0,0}\rangle +{{\rm{\Phi }}}_{1}|g\mathrm{,1,0}\rangle +{{\rm{\Phi }}}_{2}|g\mathrm{,0,1}\rangle .$$

Here, Φ_0_ is the probability amplitude of state $|e,0,0\rangle$ which corresponds to the situation when there is no emission in either of the cavities. Similarly, Φ_1_ (Φ_2_) is related to a photon emission in cavity 1 (2). The use of Eqs (1 and 2), enable us to construct the following coupled equations^11^

3 $$\begin{array}{rcl}i\hslash \frac{\partial {{\rm{\Phi }}}_{0}}{\partial t} & = & -\frac{{\hslash }^{2}}{2m}\frac{{\partial }^{2}{{\rm{\Phi }}}_{0}}{\partial {z}^{2}}+\hslash {\chi }_{1}{u}_{1}(z){{\rm{\Phi }}}_{1}+\hslash {\chi }_{2}{u}_{2}(z){{\rm{\Phi }}}_{2},\\ i\hslash \frac{\partial {{\rm{\Phi }}}_{1}}{\partial t} & = & -\frac{{\hslash }^{2}}{2m}\frac{{\partial }^{2}{{\rm{\Phi }}}_{1}}{\partial {z}^{2}}+\hslash {\chi }_{1}{u}_{1}(z){{\rm{\Phi }}}_{0},\\ i\hslash \frac{\partial {{\rm{\Phi }}}_{2}}{\partial t} & = & -\frac{{\hslash }^{2}}{2m}\frac{{\partial }^{2}{{\rm{\Phi }}}_{2}}{\partial {z}^{2}}+\hslash {\chi }_{2}{u}_{2}(z){{\rm{\Phi }}}_{0}.\end{array}$$

The set of Eq. (3) can be solved by applying boundary conditions on various interfaces of the five distinct regions as depicted in Fig. 1. It may be noted that except region V all other regions have two opposing waves. We follow the procedure of expressing Φ’s in terms of two opposite directed waves in all regions. The undetermined variables are then obtained by using continuity relations at various interfaces. Here, we consider the symmetric case of same coupling (*χ*_1_ = *χ*_2_ = *χ*) and equal lengths (*p*_2_ − *p*_1_ = *p*_4_ − *p*_3_ = *l*) for both cavities. The relative separation of the two cavities thus comes out to be *L* = *p*_3_ − *p*_2_. The transmission probability of the ultracold atoms in the excited states may be defined as

4 $${|{T}_{e}|}^{2}={|\frac{{{\rm{\Phi }}}_{0}(z={p}_{4})}{{{\rm{\Phi }}}_{0}^{inc}(z={p}_{1})}|}^{2}.$$

It is quite remarkable that the amplitude *T*_*e*_ for the two coupled cavities may be written in terms of the parameters of a single cavity^11^

5 $${T}_{e}=\frac{{t}^{2}}{\{1-exp\mathrm{(2}ikL){r}^{2}\}}.$$

Here the symbols t and r stand for the reflection and transmission amplitudes of a single cavity in the form^1^

6 $$t=\frac{1}{2}({\tau }^{+}(k)+{\tau }^{-}(k)),$$

and

7 $$r=\frac{1}{2}({\rho }^{+}(k)+{\rho }^{-}(k\mathrm{))}.$$

The parameters *τ*^±^(*k*) and *ρ*^±^(*k*) are given as

8 $${\tau }^{\pm }(k)=exp(-ikl)\,{(\cos ({k}^{\pm }l)-i{{\rm{\Sigma }}}^{\pm }\sin ({k}^{\pm }l))}^{-1},$$

and

9 $${\rho }^{\pm }(k)=i{{\rm{\Delta }}}^{\pm }\,\sin ({k}^{\pm }l)exp(ikl){\tau }^{\pm }(k),$$

such that ${k}^{\pm }=\sqrt{{k}^{2}\mp {k}_{0}^{2}}$, ${{\rm{\Sigma }}}^{\pm }=\frac{1}{2}(\frac{{k}^{\pm }}{k}+\frac{k}{{k}^{\pm }})$, and ${{\rm{\Delta }}}^{\pm }=\frac{1}{2}(\frac{{k}^{\pm }}{k}-\frac{k}{{k}^{\pm }})$. Here *k*_0_ corresponds to the (c.m.) energy which just balances the barrier’s potential (i.e., $\tfrac{\hslash {k}_{0}^{2}}{2m}=\hslash \chi$). It is useful to express width and mutual separation of the cavities in terms of the specific wave number *k*_0_ or the de Broglie wave (*λ*_*B*_ = 2*π*/*k*).

## Phase time for the resonant tunneling of ultracold atoms

In the preceding section we have shown that the transmission amplitude for ultracold atoms interacting with an arrangement of two high quality microwave cavities may be written using various parameters of a single cavity. Here we establish a relation for the phase tunneling time using the transmitted wave function corresponding to the ultraslow atoms. For an initial Gaussian distribution ($A(k)=exp(-{(k-\overline{k})}^{2}/{\sigma }^{2})$) of the incident slow atoms, we consider the situation where both cavities remain in vacuum states after tunneling of the atoms in the excited state with probability amplitude *T*_*e*_ ≡ |*T*_*e*_|*e*^*iϕ*(*k*)^. Here $\overline{k}$ corresponds to the mean momentum and *σ* represents the width of the tunneling packet. After normalization, the total transmission at *z* ≥ *p*_4_ (i.e., exit of the second cavity) may be written as

10 $$\begin{array}{rcl}|{{\rm{\Psi }}}_{T}(z,t)\rangle  & = & {\mathrm{(2}\pi )}^{-\mathrm{3/4}}\sqrt{\mathrm{2/}\sigma }\,{\int }_{-\infty }^{\infty }\,dk\,\exp (-{\sigma }^{-2}{(k-\overline{k})}^{2})\\  &  & \times {e}^{-i(\hslash {k}^{2}\mathrm{/2}m)t}|{T}_{e}|{e}^{i\varphi (k)}{e}^{ikz}|e\mathrm{,0,0}\rangle .\end{array}$$

An approximate solution of the integrand in Eq. (10) can be obtained using the Taylor expansion of the phase of *T*_*e*_. This equation gives a non zero outcome for the wave packets with small spread *σ* in a limited range around the mean momentum $\overline{k}$. At $k=\overline{k}$ the envelop ${|\langle e\mathrm{,0,0}|{{\rm{\Psi }}}_{T}(z,t)\rangle |}^{2}$ gets its peak for the maximum total phase (Θ(*k*) = *kL*_*i*_ + *ϕ*(*k*) − (*ħk*^2^/2*m*)*t*) of Eq. (10). The traversal time for the peak of the packet is then estimated by applying the stationary phase approximation (SPA)^22^

11 $${\frac{\partial {\rm{\Theta }}(k)}{\partial k}|}_{k=\overline{k}}={\frac{\partial }{\partial k}[k{L}_{i}+\varphi (k)-(\hslash {k}^{2}\mathrm{/2}m)t]|}_{k=\overline{k}}=0.$$

The phase time of tunneling is thus evaluated as

12 $${t}_{ph}={\frac{m}{\hslash k}(\frac{\partial \varphi }{\partial k}+{L}_{i})|}_{k=\overline{k}},$$

where, *L*_*i*_ = *p*_4_ − *p*_1_ is the interaction length containing lengths of both cavities and their mutual separation. It is a matter of fact that the free space traversal always corresponds to a zero phase change. Consequently, the peak of the packet will traverse a distance *L*_*i*_ (interaction length) of the free space in time ${t}_{cl}\equiv m{L}_{i}/\hslash \overline{k}$. We called it the classical traversal time as the atoms see no potential during this time span.

**Figure 2:**
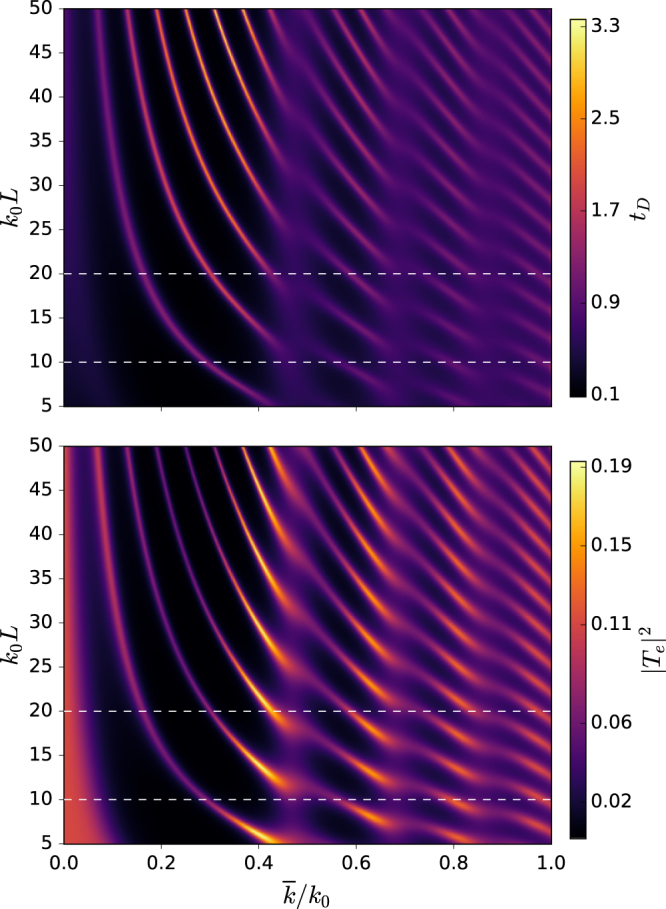
Density plot of the phase time (top-panel) and transmission probability (bottom-panel) versus average wave number $\overline{k}/{k}_{0}$ for excited state transmission. Here *k*_0_*l* = 10*π* is length of each cavity and (*k*_0_*L*) is their mutual separation.

**Figure 3:**
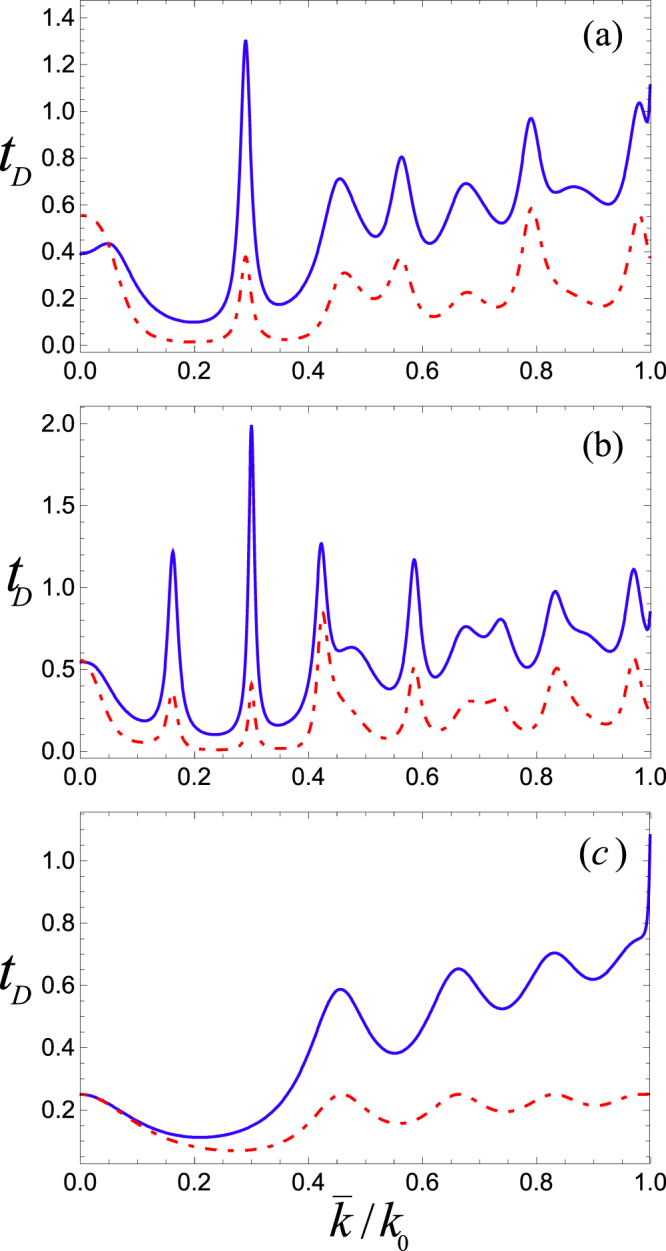
Phase time (*t*_*D*_, solid line) and transmission probability (|*T*_*e*_|^2^, broken line) vs the mean wave number. Here, length of each cavity is *k*_0_*l* = 10*π*, and the intercavity separation is (**a**) *k*_0_*L* = 10, (**b**) *k*_0_*L* = 20. The plot in (**c**) gives phase time for a single cavity.

Next, we discuss our results for the scaled phase time given by (*t*_*D*_ = *t*_*ph*_/*t*_*cl*_). Here, our primary interest is to investigate how nature of the phase time changes with various parameters of the system. The density plot shown in Fig. 2 gives dimensionless phase time and transmission probability |*T*_*e*_|^2^ versus average wave number $\overline{k}/{k}_{0}$ and the intercavity separation *k*_0_*L*. This result corresponds to the case when the incident atoms transmit in the excited state $|e\mathrm{,0,0}\rangle$ without releasing any photon in either of the two cavities. For the cavity length *k*_0_*l* = 10*π*, we obtained both subclassical (*t*_*ph*_ > *t*_*cl*_) and superclassical (*t*_*ph*_ < *t*_*cl*_) characteristics of tunneling by tuning the system’s controlling parameters. A careful look of the plot reveals that the phase time curve tracks the same pattern of resonances as exhibited by the transmission probability. For a straightforward comparison, in Fig. 3(a,b) we have shown two cross-sections of the phase time and transmission probability at different cavity separations (*k*_0_*L* = 10, *k*_0_*L* = 20) accompanied by a plot for a single cavity of the same length *k*_0_*l* = 10*π*, (See Fig. 3(c)). It must be noted that for clarity, we plot 5 × |*T*_*e*_|^2^ in Fig. 3(a,b). Here, we find that the behavior of the phase time is entirely different in comparison with the single cavity as discussed in ref.^22^, where phase time stays superclassical for the same range of the mean wave number $\overline{k}/{k}_{0}$. In contrast, for the two-cavity system, here we see an alternate sub and superclassical character of the tunneling time along the momentum axis. It is due to the increase in the number of resonances of the transmission amplitude in case of the two coupled cavities. It is interesting to mention that a similar character of the increased resonances was experimentally detected by Tsu *et al*. for the tunneling of electrons through multi barrier potentials^31^. Moreover, the effect of separation length between the two coupled cavities is quite evident from Fig. 2, making it yet another important controlling parameter of the multi-cavity system.

**Figure 4:**
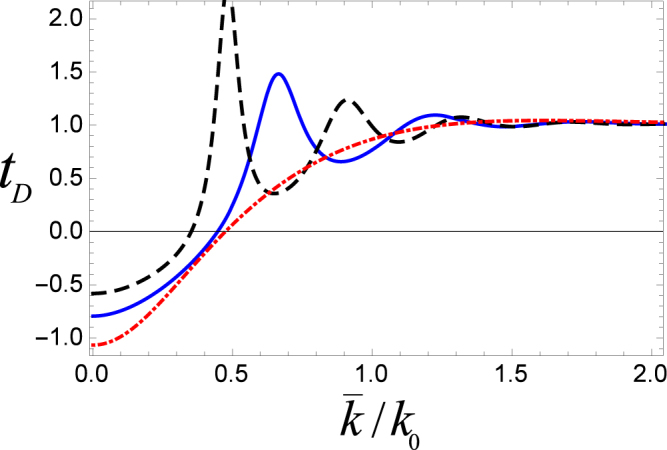
Phase time (*t*_*D*_) vs the mean wave number for a system of two cavities where length of each cavity is *k*_0_*l* = *π*/2, and the intercavity separation is *k*_0_*L* = 3 (solid blue curve), and *k*_0_*L* = 5 (large dashed black curve). The dotted dashed red curve represents phase time through a single cavity.

In Fig. 4, we plot phase time for cavity length *k*_0_*l* = *π*/2, separation lengths *k*_0_*L* = 3 (solid blue curve) and *k*_0_*L* = 5 (large dashed black curve). For the sake of instant comparison, the dotted dashed red curve representing phase time for a single cavity system as examined in^22^ is also plotted. Here, for the double cavity case, we obtained both super and subclassical values for the phase time of ultracold atoms which is not the case for a single cavity where only superclassical values were found as indicated by the dotted dashed curve. In our model, the intercavity separation may be exploited for obtaining subclassical peaks of even higher magnitude i.e., the atoms while tunneling through the coupled cavities are further slow down during the interaction process which is a sort of cooling effect in the multi-cavity arrangement of the present study.

**Figure 5:**
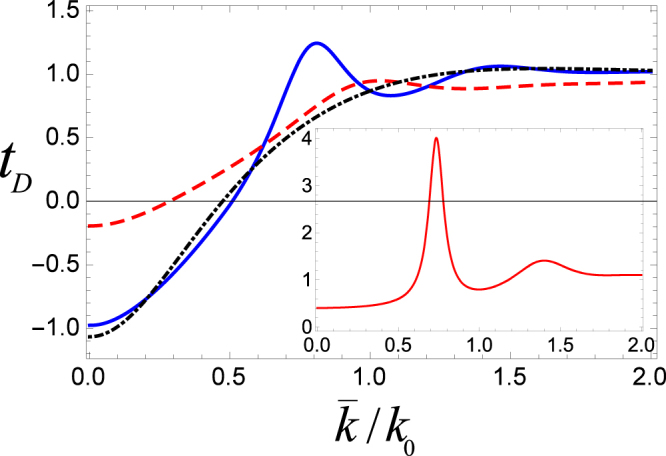
Phase time (*t*_*D*_) vs the mean wave number such that the blue solid (black dotted dashed) curve represents phase time through a system of double (single) cavity. The red dashed curve is for a double well while the inset is for a double barrier potential. Length of each cavity is taken to be *k*_0_*l* = *π*/2, and the intercavity separation is *k*_0_*L* = 2.

Next, in Fig. 5, we compare the results of our model (i.e., phase time of ultracold atoms through double cavities) with the tunneling through double barrier and double well potentials. It is useful to mention that in case of double barriers and double well the transmission amplitudes are written as

13 $${B}_{k}=\frac{{\tau }^{+2}(k)}{1-exp\mathrm{(2}ikL){\rho }^{+2}(k)},$$

and

14 $${W}_{k}=\frac{{\tau }^{-2}(k)}{1-exp\mathrm{(2}ikL){\rho }^{-2}(k)}.$$

In the result of Fig. 5, other important parameters are the cavity (barrier, well) length *k*_0_*l* = *π*/2 and the intercavity (barrier, well) separation *k*_0_*L* = 2. Here, the blue solid (red dashed) curve represents the phase time through double cavities (double well) potential. The black dotted dashed curve is for a single cavity while the inset is for a double barrier potential. The phase time for double barrier potential has only positive values with both super and subclassical nature. Here, the subclassical peak is much larger than the one obtained for the double cavity system. On the other hand, for a double well potential, although *t*_*D*_ may take positive as well as negative values but they always remain superclassical. Interestingly, phase time characteristics for the two coupled cavity arrangement in our case exhibit all different features that are individually shown by the double barrier or double well systems. It is clearly indicated by the solid blue curve where the phase time for the double cavity system has both negative/positive and superclassical/subclassical values. It is because microwave cavities may exhibit a simultaneous barrier and well type nature which is quite unique. It can be noted that for the given parameters, behavior of the phase time for a single cavity is somehow similar to the double well potential as both are superclassical only with positive and negative values. Nevertheless, the obvious disparity in this case is the magnitude of the negative value which is much smaller for the double well potential. Phase time being negative is a superclassical behavior which is equivalent to the situation where peak of the packet emerges at the other end even before its entrance into the interaction region. The reason for this seems to be the interference of the two opposing waves associated with the tunneling atoms (See for details^37,38^). Further, as evident from Eq. (12), phase time gets negative values only when the slope $(\frac{\partial \phi }{\partial k})$ is negative and it has a larger absolute value than *L*_*i*_. In some sense, the negative phase time in our case resembles to the negative group velocities (−*v*_*g*_) of the electromagnetic (EM) pulses propagating in a dielectric medium^39^.

**Figure 6:**
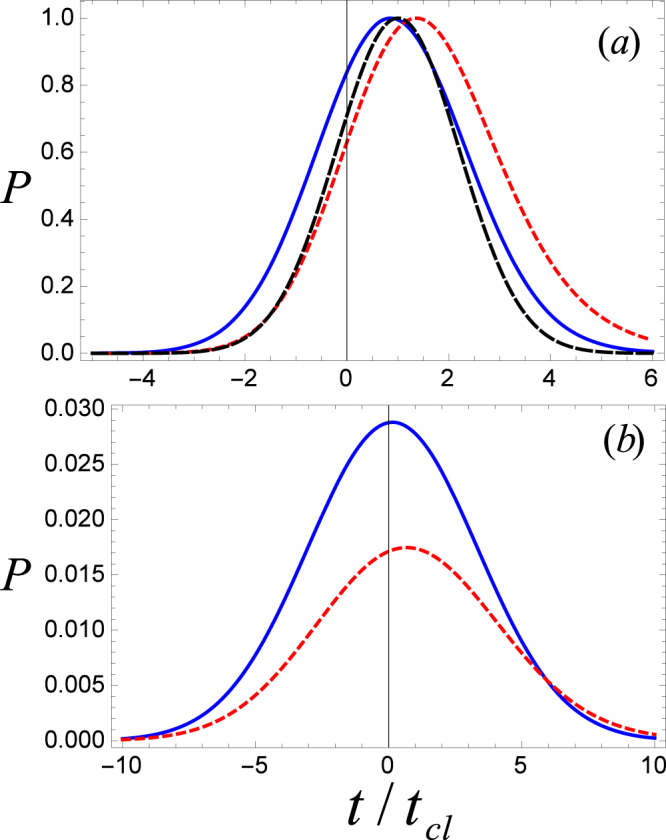
Panel (a) shows probability density P at *z* = *p*_4_ versus phase time *t*_*D*_ for cavity length *k*_0_*l* = 10*π*. The solid blue (small dashed red) line gives P when atoms transmit through double cavities with *k*_0_*L* = 10(20). The large dashed black curve is for propagation of the packet through an equal length (*L*_*i*_) of the free space. Panel (b) gives a comparison of the wave packet tunneling through a system of two cavities (dashed red curve) with a single cavity (solid blue curve).

In the compilation of the above results we used the stationary phase approximation. Next, we want to make sure about the validity of this approximation in our problem. For this purpose, we plot the probability density $(P\equiv {|\langle e\mathrm{,0,0}|{{\rm{\Psi }}}_{T}(z,t)\rangle |}^{2}/\sigma )$ at *z* = *p*_4_ verses *t*_*D*_ for *k*_0_*l* = 10*π* while solving Eq. (10) exactly without any approximation (See Fig. 6). The solid blue (small dashed red) curve in Fig. 6(a) represents P after transmission through double cavities with separation length *k*_0_*L* = 10 (20), mean wave number $\overline{k}/{k}_{0}=0.299$, and width of the packet *σ*/*k*_0_ = 0.01 (all curves normalized to unity). It is clear that for the intercavity separation *k*_0_*L* = 10, peak of the packet is at *t*_*D*_ = 0.86 (a superclassical value) which is the same as given by Fig. 3(a) at the mean wave number 0.299 using the technique of SPA. Hence, the exact solution of *t*_*D*_ obtained by a numerical integration matches perfectly with the one resulted from the approximation (SPA). Similarly, for *k*_0_*L* = 20, tunneling of the wave packet is subclassical as indicated by the small dashed red curve where the peak of the packet lies at *t*_*D*_ = 1.4. The large dashed black curve is for propagation length (*L*_*i*_) of the free space which peaks at unity showing that the phase tunneling time and the classical time are the same for the free space propagation. Panel (b) gives a comparison of the wave packet tunneling through a system of two cavities (dashed red curve) with a single cavity (solid blue curve). Obviously, the probability density in case of double cavities is much smaller then a single cavity.

## Conclusions

In this study, we explore the tunneling characteristics of ultraslow atoms for a system of two high-*Q* Mazer cavities. For this purpose, we considered a beam of the initially excited atoms interacting with an arrangement of two microwave cavities. Such an interaction between ultraslow atoms and vacuum cavity modes resembles to a scattering like phenomenon with some nonzero reflection and transmission amplitudes. In order to analyze various properties of the tunneling time of ultraslow atoms, we mainly focussed on the transmission part of the scattering by evaluating transmission amplitude of the interacting atoms. A relation for the phase tunneling time is obtained by solving the integral in the transmitted wave function (Eq. (10)) with the help of the stationary phase condition. Here, we find that phase time of tunneling for the ultraslow atoms is remarkably modified with the length of each cavity and their relative separation. Further, it is noted that for the double cavity system the transmission amplitude exhibits fast resonances which results in the alternate sub and superclassical character of the phase tunneling time. A similar result of increased resonances was observed in the experiment of Tsu and coworkers related to the tunneling of electrons through multibarrier potentials of a finite superlattice^31^. This confirms the enhanced resonances to be a typical feature of a multi-potential arrangement. We compare the tunneling of atoms through double cavities with the tunneling through double barrier and double well arrangements. It is found that the phase time characteristics through double cavity arrangement contain all properties which are individually exhibited by the double barrier and double well potentials. It is due to the fact that the microwave cavity simultaneously behaves like a barrier and well to the incident slow atoms which is quite unique. Moreover, we noticed that position and amplitudes of the subclassical peaks in the phase time curves might be smartly repositioned with the help of the intercavity separation parameter *k*_0_*L*. Interestingly, exact solution of the numerical integration is found to be perfectly matched with those given by the stationary phase approximation.
